# Longitudinal beta regression models for analyzing health-related quality of life scores over time

**DOI:** 10.1186/1471-2288-12-144

**Published:** 2012-09-17

**Authors:** Matthias Hunger, Angela Döring, Rolf Holle

**Affiliations:** 1 Helmholtz Zentrum München, German Research Center for Environmental Health (GmbH), Institute of Health Economics and Health Care Management, Ingolstädter Landstr 1, Neuherberg, 85764, Germany; 2 Helmholtz Zentrum München, German Research Center for Environmental Health (GmbH), Institute of Epidemiology II, Ingolstädter Landstr. 1, Neuherberg, 85764, Germany

**Keywords:** Health-related quality of life, Beta regression, Longitudinal study, Mixed model, Marginal model

## Abstract

**Background:**

Health-related quality of life (HRQL) has become an increasingly important outcome parameter in clinical trials and epidemiological research. HRQL scores are typically bounded at both ends of the scale and often highly skewed. Several regression techniques have been proposed to model such data in cross-sectional studies, however, methods applicable in longitudinal research are less well researched. This study examined the use of beta regression models for analyzing longitudinal HRQL data using two empirical examples with distributional features typically encountered in practice.

**Methods:**

We used SF-6D utility data from a German older age cohort study and stroke-specific HRQL data from a randomized controlled trial. We described the conceptual differences between mixed and marginal beta regression models and compared both models to the commonly used linear mixed model in terms of overall fit and predictive accuracy.

**Results:**

At any measurement time, the beta distribution fitted the SF-6D utility data and stroke-specific HRQL data better than the normal distribution. The mixed beta model showed better likelihood-based fit statistics than the linear mixed model and respected the boundedness of the outcome variable. However, it tended to underestimate the true mean at the upper part of the distribution. Adjusted group means from marginal beta model and linear mixed model were nearly identical but differences could be observed with respect to standard errors.

**Conclusions:**

Understanding the conceptual differences between mixed and marginal beta regression models is important for their proper use in the analysis of longitudinal HRQL data. Beta regression fits the typical distribution of HRQL data better than linear mixed models, however, if focus is on estimating group mean scores rather than making individual predictions, the two methods might not differ substantially.

## Background

Health-related quality of life (HRQL) has become an increasingly important outcome parameter in clinical trials and epidemiological research to support clinical and policy decision making or to monitor population health [1,2]. Treatment effects on HRQL and population values are commonly estimated using regression techniques, however, HRQL scores typically exhibit specific properties that make the use of ordinary least square (OLS) regression at least doubtful for such kind of data [3,4]. In particular, they are continuous variables bounded at both ends of the distribution (e.g. at 0 and 1) and are often highly skewed. As a consequence, several alternative regression methods have been suggested such as censored least absolute deviation models [5], Tobit models [4,5] and median regression [5,6]. A regression technique that is gaining increasing attention in the analysis of doubly bounded outcome measures is the beta regression as introduced by Ferrari and Cribari-Neto [7]. Beta regression was first mainly used in economic and psychological applications [8,9], but has recently also been proposed to analyze generic HRQL [3,10]. In these contributions, it was shown that beta regression can have substantial advantages over OLS regression, especially in estimating covariate effects when the true incremental effect is large [3]. However, they also revealed that beta regression may perform poorly in handling observations on the boundary points [10].

While several regression models have been suggested to address the idiosyncrasies of HRQL data in a cross-sectional design, research on longitudinal regression models is less well developed. This is both surprising and unfortunate given that change in HRQL over time is often the primary interest in applied work. Currently, longitudinal quality of life data are mostly analyzed using change scores [11], repeated measures ANCOVA [12,13], and linear mixed models (LMM) [14,15].

Beta regression has recently been expanded to deal with longitudinal data by introducing a beta-distributed generalized linear mixed model (GLMM) [16,17]. However, a more-in-depth-comparison with traditionally employed methods, especially the LMM, is still lacking. Also, to date no study has examined the applicability of longitudinal beta regression models to analyze HRQL scores over time.

An elaborate comparison between beta regression and linear regression in a longitudinal design is not only important with respect to model fit and predictive ability. It is also important to realize that in longitudinal models with non-identity link such as beta regression, the interpretation of parameter coefficients depends on how the correlation between observations is accounted for [18]. Basically, two different approaches can be distinguished: A subject-specific approach as implemented by the GLMM, and a population-averaged approach using marginal models [19].

The purpose of this study was to examine the use of beta regression methods to analyze longitudinal HRQL data. We describe the conceptual differences between mixed effect models and marginal models researchers should be aware of when extending beta regression to the longitudinal case. Using two empirical datasets with both generic and disease-specific HRQL scores, we compare estimated effects and predictive accuracy of the beta regression methods to those of the commonly used LMM.

## Methods

### Empirical data sets

We fitted longitudinal regression models to two empirical data sets representing different distributional features typically encountered when analyzing HRQL scores in practice. Data in the first example come from a cohort study, while data in the second example were collected alongside a randomized controlled trial (RCT). In both cases, we examined HRQL scores over time with respect to two groups of individuals.

In the first application, we examined how the generic SF-6D health utility index changed over a 7-year period in an older general population sample. Data come from the population-based KORA S4/F4 cohort study conducted in the region of Augsburg in Southern Germany. The sample used in our analyses involved 1225 subjects aged 60 years and above recruited for the S4 survey in 1999. In 2006–2008, 812 of these 1225 subjects took part in the follow-up study F4. A detailed description of study design, sampling method and data collection can be found elsewhere [10,20]. Besides other questions, individuals were asked at both time points if they have diabetes mellitus. Also, subjects answered the 12-item Short-Form Health Survey (SF-12), from which the SF-6D utility index was derived [21]. Health utilities can be used to calculate quality-adjusted life-years (QALYs) and usually range between 0 (health state similar to death) and 1 (‘perfect health’). However, due to the specific health state classification behind the SF-6D, possible values only lie between 0.345 and 1 [21]. Focus in this analyses was on the question how diabetes mellitus is associated with HRQL over time.

The second application investigated disease-specific HRQL in stroke patients over time, measured by the Stroke Impact Scale (SIS) [22]. Data were collected alongside an RCT evaluating a patient education programme for stroke survivors in neurological rehabilitation based on the conceptual framework of the International Classification of Functioning, Disability and Health (ICF). The study sample comprised 212 patients in the age range of 22 to 83 years recruited between 2008 and 2009 in seven rehabilitation clinics in Germany. Details on clinical characteristics and data collection methods can be found elsewhere [23]. Patients answered self-report questionnaires before and after the education programme (median difference 10 days) as well as at a postal follow-up conducted 6 months later. At post-intervention and follow-up, questionnaire data were available for 183, and 171 patients, respectively. Patients in the sample were assigned to two different rehabilitation phases (C and D), following the six-phase model of the German Federal Rehabilitation Council. The distinction between phase C and D contrasts patients still dependent on a high degree of nursing and medical care to those having mostly gained independence in the activities of daily life [24]. Since regaining mobility is a major goal of post-stroke rehabilitation, the objective of the analyses was to analyze SIS mobility subscale (SIS-Mob) scores over time. SIS-Mob scores range from 0 to 100, with higher values indicating better HRQL. We divided scores by 100 in order to make them fit to the support of the beta distribution. In this analysis, we focused on the comparison of time trends between patients in phase D and those in phase C but ignored whether patients were assigned to the intervention or to the control group.

Both studies were approved by the local ethic committee.

### Beta regression

#### Beta regression for cross-sectional data

The beta distribution is a continuous probability distribution defined over the unit interval with density function

$$\;f\left(y;\mu ,\phi \right)=\frac{\Gamma \left(\phi \right)}{\Gamma \left(\mu \phi \right)\Gamma \left(\left(1-\mu \right)\phi \right)}\;y^{\;\mu \phi -1}{\left(1-y\right)}^{\left(1-\mu \right)\phi -1},0<y<1\text{,}$$

where *Γ* (.) denotes the gamma function [7]. The parameter *μ* denotes the expected value of *Y*, i.e. E(*Y*) = *μ*. The parameter *ϕ* fulfils the definition of a precision parameter since – for fixed *μ* – the greater the value of *ϕ*, the smaller the variance of the dependent variable. More specifically,

$$\;Var\left(Y\right)=\frac{\mu \left(1-\mu \right)}{1+\phi }\text{.}$$

The beta distribution is part of the exponential family, but not of canonical form [18,25]. In beta regression models, the mean parameter *μ* ∈ (0,1) of the beta distribution is expressed as a function of covariates, while the precision parameter *ϕ* ∈ ℝ^+^ is treated as nuisance. To map the linear predictor into the space of observed values on the unit interval, the logit link

(1) $$\;g\left(\mu_{i}\right)=\log\frac{\mu_{i}}{1-\mu_{i}}=x_{i}^{T}\beta \text{,}$$

is commonly used as the link of choice where $x_{i}^{T}$ denotes a vector of covariates, and *β* refers to the vector of regression coefficients, *i* = 1,…,*N*[8,26]. The beta distribution is defined on the open unit interval only. If ones and zeros are observed, these values need to be transformed in order to fall into the open unit interval (0,1). This can be achieved by either minimally compressing the entire range of observed values, or by only transforming the boundary points to slightly smaller or greater values, respectively. The most frequently applied transformation is given by

(2) $$\;Y^{*}=\left[Y\left(N-1\right)+0.5\right]/N$$

where Y^*^ is the transformed and *Y* is the untransformed dependent variable [8,17]. Alternatively, it has been suggested to add a small amount ε, e.g. 0.005 or 0.01 to the lower bound, and to subtract the same amount from the upper bound [8,16]. A reasonable choice involves the following trade-off: On the one hand, large values for ε shrink the data more toward 0.5 and may bias the estimates toward no effect; on the other hand, moving zero- and one-valued observations an insufficient distance away from the boundary may lead to instable estimates because this can cause the likelihood to have a local or even global mode in this area [16,27]. Hunger *et al.* also observed that when the resulting values are too close to the boundary points, precision of the estimates may appreciably decrease [10]. Therefore, it has been recommended to use sensitivity analyses in order to check whether different endpoint handling methods affect parameter estimates [8,16].

#### Beta GLMM for longitudinal data

In longitudinal analyses or in the case that subjects are clustered within sampling units or geographical entities, measurements within the same person or unit are typically correlated, violating the assumption of conditionally independent observations in regression models [18]. One possibility to account for these dependencies is to add random cluster or subject effects into the linear predictor. Without loss of generalizability, consider the case of longitudinal designs where *j* = 1,…,*n*_*i*_ observations are nested within *i* = 1,…,*N* subjects. Let *b*_*i*_ denote a vector of subject-specific random effects for individual *i*.

In the linear regression model, the inclusion of random effects leads to the LMM given by

(3) $$Y_{\mathrm{ij}}=x_{\mathrm{ij}}^{T}\beta +z_{\mathrm{ij}}^{T}b_{i}+\varepsilon_{\mathrm{ij}}\;\text{with}\;b_{i}∼N\left(0,G\right)\;\text{and}\;\varepsilon_{\mathrm{ij}}∼N\left(0,\sigma^{2}\right)$$

Similarly, adding random effects to the beta regression model in (1) yields the beta GLMM [16,17] given by

(4) $$\;\log\frac{\mu_{\mathrm{ij}}}{1-\mu_{\mathrm{ij}}}=x_{\mathrm{ij}}^{T}\beta +z_{\mathrm{ij}}^{T}b_{i}\;\text{with}\;b_{i}∼N\left(0,G\right)\text{.}$$

In both cases, $z_{\mathrm{ij}}^{T}$is a vector of covariates, and *G* denotes the positive definite covariance matrix of the random effects. Note that although the assumption of normality for the random effects is common and statistically convenient, other distribution assumptions are possible in principle [17]. In a longitudinal design, *b*_*i*_ typically is a scalar (for random intercept only models) or a bivariate vector (for models with random intercept and random slope). In the first case, *z*_*ij*_ = 1, while in the second case, $z_{\mathrm{ij}}^{T}=\left(1,t_{\mathrm{ij}}\right)\text{,}$ where *t*_*ij*_ is the time of measurement *j* for subject *i*. Models with random slope allow the linear effect of time to vary across subjects. Model parameters are estimated by maximizing the marginal likelihood which is obtained by integrating out the unobserved random effects *b*_*i*_ from the likelihood function [16].

Although the inclusion of random effects in the beta GLMM is conceptually the same as in the LMM, there are important implications with regard to the interpretation of regression parameters: In the LMM, the fixed effects have both a subject-specific (together with the random effects *b*_*i*_) and a population-average interpretation. This follows directly from (3) because

$$\;E\left(Y_{\mathrm{ij}}|b_{i}\right)=x_{\mathrm{ij}}^{T}\beta +z_{\mathrm{ij}}^{T}b_{i},\;\text{and}\;E\left(Y_{\mathrm{ij}}\right)=x_{\mathrm{ij}}^{T}\beta \text{.}$$

In the beta GLMM, however, the regression parameters only have a subject-specific interpretation and no longer describe the effect of the respective variable on the population in general [18]. This is due to the non-linear transformation of the mean response (i.e. the logit link) since it can be deduced from (4) that

$$\;\begin{array}{c} \text{logit}\left(E\left(Y_{\mathrm{ij}}|b_{i}\right)\right)=x_{\mathrm{ij}}^{T}\beta +z_{\mathrm{ij}}^{T}b_{i},\\ \;\text{but logit}\left(E\left(Y_{\mathrm{ij}}\right)\right)\neq x_{\mathrm{ij}}^{T}\beta \text{.} \end{array}$$

This individual-specific interpretation means, for example, that the parameter coefficient of the covariate ‘diabetes’ in the first empirical application refers to the difference in mean SF-6D scores on the logit scale between an individual with diabetes and the same individual supposed not to have diabetes [28].

#### Beta GEE

If a population-averaged interpretation of the regression coefficients is desired, for example the mean difference between the groups of individuals with and without diabetes, an alternative to the beta GLMM is the marginal model. The term ‘marginal’ means that the mean response modeled is conditional only on covariates and not on other responses or random effects [18].

Marginal models do not specify the full joint distribution of the data, but only specify a mean function, a variance function, and a correlation structure between observations within one individual. Mean and variance function (in some models together with an additional scaling factor *ϕ*) are often suggested by the canonical form of the exponential family [29]. For the beta distribution, it is convenient to specify $\log\frac{\mu_{\mathrm{ij}}}{1-\mu_{\mathrm{ij}}}=x_{\mathrm{ij}}^{T}\beta$ following (1), and using the variance function $\text{Var}\left(Y_{\mathrm{ij}}|x_{\mathrm{ij}}\right)=\phi \mu_{\mathrm{ij}}\left(1-\mu_{\mathrm{ij}}\right)$.

Note that this specification of mean and variance structure is also commonly used in GEE models to analyze binary data. The only difference is the additional scaling parameter *ϕ* which is usually not used in a GEE for binary data. The inclusion of the scaling parameter in the beta GEE has no impact on the estimation of the mean model parameters, however, it has the advantage that large estimates for *ϕ* can indicate heterogeneity in the data that is not accounted for by the model [3]. Similarities also exist to the inclusion of an additional dispersion parameter in quasi-binomial models for cross-sectional data and such methods have already been used in literature to model HRQL scores [30-32]. For the working correlation matrix, several choices are possible. Among them, compound symmetry, autoregressive structure, and unstructured correlation are most commonly used in longitudinal analyses [18]. Variance function and correlation matrix can then be combined into a ‘working’ covariance matrix *V*_*i*_. Parameter estimates in the marginal model are obtained by solving the Generalized Estimating Equations (GEE) introduced by Liang and Zeger [33,34].

$$\;\sum_{i=1}^{N}D_{i}^{T}V_{i}^{-1}\left(Y_{i}-\mu_{i}\right)=0$$

where $Y_{i}={\left(Y_{i1},\ldots ,Y_{in_{i}}\right)}^{T},\;\mu_{i}={\left(\mu_{i1},\ldots ,\mu_{in_{i}}\right)}^{T}\text{,}$ and $D_{i}=D_{i}\left(\beta \right)=\partial \mu_{i}\left(\beta \right)/\partial \beta^{T}$.

In general, there are no closed-form solutions, so that iterative algorithms are used. Specific types of GEEs can further be distinguished according to how the covariance parameters are estimated. While the early contributions on GEEs mainly used the methods of moments, other approaches using pseudo-likelihood techniques and quadratic estimation equations methods have also been suggested [35]. The latter approach, for example, is implemented in the SAS GLIMMIX procedure. Parameter coefficients in the GEE are estimated consistently even if the covariance structure is mis-specified, however, a careful choice of the working correlation may improve efficiency of the estimates. Valid standard errors for $\hat{\beta }$ can be calculated by using the so called sandwich estimator [18]. Since the full likelihood of the data is not specified in GEE models, likelihood-based criteria to assess model fit are not available.

#### Missing data

Missing data are an important issue in many quality of life studies. Whether inference remains valid in the case of incomplete data depends on the underlying missing data mechanism and the statistical methods used. Estimates from the beta GLMM remain valid if the data are missing at random (MAR), i.e. that given the observed data, the probability of a missing observation does not depend on the unobserved data [36,37]. However, this requires maximum likelihood estimation based on adaptive Gaussian quadrature to be used; other estimation methods such as penalized quasi likelihood (PQL) can lead to biased estimates of the covariate effects [18]. In contrast, inferences with the beta GEE are only valid under the stronger assumption that data are missing completely at random (MCAR), i.e. that missingness is independent of both, unobserved and observed data [33,38]. Extensions of the GEE have been proposed to allow the data to be MAR, however, these methods either focus on monotone missing patterns or require the correct specification of the working correlation matrix [39,40].

#### Model comparison and residuals: current state of research

Model comparison and model checking in the GLMM and GEE framework is not straightforward and suitable methods are sparse [41]. In general, if GLMMs are estimated using a full likelihood approach, models can be compared using information criteria such as Akaike Information Criterion (AIC) or Bayesian Information Criterion (BIC) [16]. AIC and BIC are measures of the likelihood, penalized for the complexity of the model. Zimprich suggested comparing beta GLMM and LMM on the basis of a pseudo-R^2^ which is motivated from the pseudo-R^2^ suggested by Cox and Snell for model comparison in logistic regression models [17,42]. It is defined as

$$\;R^{2}=1-{\left\{\frac{L_{\mathrm{Intercept}}}{L_{\mathrm{Full}}}\right\}}^{\frac{2}{N}}$$

where *L*_*Intercept*_ is the likelihood of a simple intercept-only linear model fit to the data, *L*_*Full*_ is the likelihood of the considered beta GLMM or LMM, and *N* is the total number of observations. The pseudo-R^2^ compares the likelihood of the observed data in the beta GLMM and LMM with that of a simple intercept-only linear regression model. Thus, it reflects the improvement each model has over a model without explanatory variables and can be interpreted as the geometric mean squared improvement per observation [42].

There are two types of residuals in the GLMM. Depending on the level on which fitted values are produced, one can distinguish average Pearson residuals (related to the unconditional mean ${\text{g}}^{-1}\left(x_{\mathrm{ij}}^{T}\beta \right)$) and individual-specific residuals (relating to the conditional means ${\text{g}}^{-1}\left(x_{\mathrm{ij}}^{T}\beta +z_{\mathrm{ij}}^{T}b_{i}\right)$) where g denotes the link function of the regression model (i.e. the logit in the beta GLMM). Diagnostic plots typically use individual-specific residuals. Several different residuals have been proposed for use in beta regression with independent observations, namely standardized residuals, deviance residuals, weighted residuals, and standardized weighted residuals [7,43]. However, none of these residuals has yet been extended to be applicable in the mixed regression context.

Basu and Manca used a beta regression model to analyze QALY data and examined raw scale residuals to evaluate goodness of fit [3]. In particular, they calculated mean residuals across deciles of the linear predictor in order to identify systematic patterns of misfit in the predictions.

### Model specification

In both empirical examples, we compared the performance of LMM, beta GLMM, and beta GEE model. Response variables were the SF-6D score and the SIS mobility subscale (SIS-Mob) score, respectively.

We transformed the zero- and one-valued responses in our empirical datasets to 0.005 and 0.995, respectively [8,10,16]. This is because transformation (2) depends on the number of observations, and its use in the large KORA data would move the one-valued observations to 0.9997 which is extremely close to the upper bound. However, to ensure that estimates are not affected by this choice, we also used other values for ε between 0.002 and 0.01 to move observations away from the boundary points.

Covariates in the regression models were age at baseline, sex, and time point. In the KORA data, we additionally included diabetes and its interaction with time. Thus, the respective beta GLMM was given by

$$\;\begin{array}{cc} \log\frac{\mu_{\mathrm{ij}}}{1-\mu_{\mathrm{ij}}} & =\beta_{0}+\beta_{1}age_{i}+\beta_{2}sex_{i}+\beta_{3}time_{\mathrm{ij}}\\ & \;+\beta_{4}diab_{\mathrm{ij}}+\beta_{5}diab_{\mathrm{ij}}\times timeII_{\mathrm{ij}}+b_{i}\text{,} \end{array}$$

where *timeII*_*ij*_ is the dummy variable for the second measurement time.

In the ICF stroke data, we additionally included rehabilitation phase and its interaction with time:

$$\;\begin{array}{cc} \log\frac{\mu_{\mathrm{ij}}}{1-\mu_{\mathrm{ij}}} & =\beta_{0}+\beta_{1}age_{i}+\beta_{2}sex_{i}+\beta_{3}timeII_{\mathrm{ij}}\\ & \;+\beta_{4}timeIII_{\mathrm{ij}}+\beta_{5}phaseD_{i}+\beta_{6}phaseD_{i}\\ & \;\times timeII_{\mathrm{ij}}+\beta_{7}phaseD_{i}\times timeIII_{\mathrm{ij}}+b_{i} \end{array}$$

where *timeII*_*ij*_ and *timeIII*_*ij*_ are the dummy variables for the second and third measurement times, respectively. The same (fixed effects) covariate structure was specified for the beta GEE models.

Since we only had two to three time points, our mixed models only contained a random intercept but no random slope component. In accordance, we chose a compound symmetry correlation structure in the GEE models, assuming that all measurements on the same unit are equally correlated. In the case of two measurements only, this structure is identical to more complicated structures such as autoregressive correlation.

Taking an individual-specific perspective, we compared model fit of LMM and beta GLMM using AIC, BIC, and pseudo-R^2^. However, in contrast to Zimprich, we did not calculate the pseudo-R^2^ by comparing the likelihood of the models specified above to that of an intercept-only linear model, but to the likelihood of a simple LMM with random intercept only. This is because for longitudinal data, the correlation between observations within the same individual should also be accounted for in the basic model used for comparison.

To further examine whether the two models provide a good fit to all parts of the data, we calculated mean raw residuals on the individual-specific level across deciles of the corresponding linear predictor [3]. If these means are not randomly scattered around 0, this indicates a systematic misfit of the model.

Taking the population average perspective, we compared the unconditional predictions from the LMM with the corresponding predictions from the marginal beta GEE model. Fore each time point we calculated adjusted mean HRQL scores stratified by diabetes (in the KORA data) or rehabilitation phase (in the ICF stroke data).

If an individual had a missing quality of life score or missing covariates at a certain time point, we deleted the respective observation but did not exclude the entire individual from the analysis.

All models were estimated using the GLIMMIX procedure in SAS. We approximated the marginal likelihood in the beta GLMM through Gaussian quadrature which is implemented in the SAS GLIMMIX procedure (from version 9.2) by the method = quad option. The code used to fit LMM, beta GLMM and beta GEE to the KORA data is provided in Additional file 1.

## Results

In the KORA data, 91 observations were deleted due to missing values in the response variable. One additional observation was removed due to missing information on the diabetes status. This reduced the final sample size from 2037 to 1945. In the stroke data, the observations from 15 participants were deleted because they had no information on the rehabilitation phase. Nine further observations were removed due to missing values in the response variable. This reduced the final size from 566 to 517. In the KORA data, mean age at baseline was 66.2 years (SD 4.3), and 592 (50.9%) participants were male. The percentage of individuals with diabetes was 8.9% at baseline and 16.2% at follow-up. Mean age in the ICF stroke data was 57.2 years (SD 12.8), and 115 (54.3%) individuals were male. About two third (67.5%) of the participants were assigned to rehabilitation phase D, and one third to phase C.

**Figure 1:**
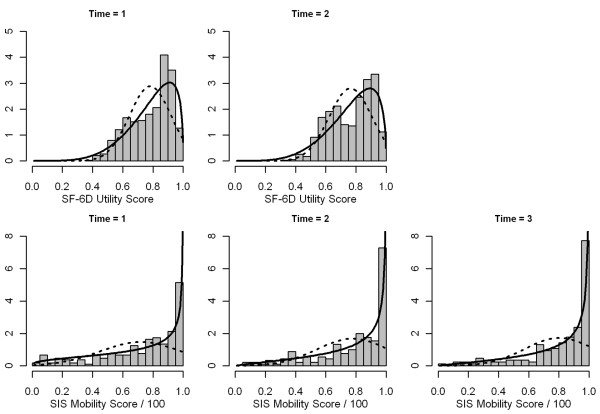
**Distribution of SF-6D utility scores by time in the KORA data (upper part) and distribution of the SIS Mobility scores by time in the ICF stroke data (lower part).** The curves represent estimated single density functions of the beta (solid) and the normal (dashed) distribution fitted to the univariate data.

When single density curves were fitted to the univariate data, in both examples, the beta distribution reproduced the shape of the observed HRQL score (SF-6D and SIS-Mob) distributions clearly better than the normal distribution (Figure 1). It accommodated the left-skew of the observed data and respected the boundary points while large parts of the fitted normal density function were lying outside the theoretically possible range of HRQL values, especially in the ICF stroke data.

The parameter estimates of the regression analyses fitted to the KORA data, are shown in Table 1. Comparing LMM and beta GLMM, one observes that age, sex and diabetes had a significant effect on the mean SF-6D utility score in both models, however, with an AIC of −2723 and a BIC of −2682, the beta GLMM fitted the observed data better than the LMM (AIC −2441; BIC −2401). This is also reflected by the pseudo-R^2^ statistics. (0.054 in the LMM, 0.181 in the beta GLMM).

**Table 1:** Parameter estimates of LMM, beta GLMM and beta GEE in the KORA data (N = 1945)

**Parameter coefficients**	** LMM**		** Beta GLMM**		** Beta GEE**	
	**Estimate**	**p value**	**Estimate**	**p value**	**Estimate**	**p value**
Intercept	0.7808	<0.0001	1.3534	<0.0001	1.2816	<0.0001
Age at baseline (centered)	−0.0036	<0.0001	−0.0185	0.0007	−0.0209	<0.0001
Male sex	0.0525	<0.0001	0.3483	<0.0001	0.3000	<0.0001
Time	−0.0140	0.0002	−0.0788	0.0004	−0.0815	0.0002
Diabetes	−0.0267	<0.0001	−0.1538	0.0002	−0.1586	<0.0001
Diabetes*Time	−0.0300	0.0544	−0.1837	0.0608	−0.1513	0.0813
σ^2^	0.0091					
ϕ			14.45			
**Variance of random effects**	Estimate	SE	Estimate	SE	Estimate	SE
Variance	0.0095	0.0007	0.3854	0.0309		
**Covariance estimates**						
Variance					0.0536	0.0028
Compound symmetry					0.0564	0.0042
Scale					0.0240	
**Fit statistics**						
−2LogL	−2457		−2739		-	
AIC	−2441		−2723		-	
BIC	−2401		−2682		-	
Pseudo-R^2^†	0.0535		0.1812		-	

*LMM* Linear mixed model, *GLMM* Generalized linear mixed model, *GEE* Generalized estimating equations, *SE* Standard error, *CS* Compound Symmetry, *AIC* Akaike information criterion. *BIC* Bayesian information criterion.

†Compared to linear random-intercept model with -2LogL = −2350.

Interpretation of parameter estimates in the beta regression model is similar to logistic regression where exponentiated coefficients can be interpreted in terms of odds ratios. For example, the parameter coefficient of male sex in the beta GLMM means that for a man, the ratio between the expected quality of life score *μ* and the difference to perfect health (1-*μ*) is about exp(0.3483) = 1.42 times higher than for a woman with the same set of covariates (and random effect).

The interaction between diabetes and time suggests that the decline in HRQL over time was slightly larger in individuals with diabetes, however, the interaction term was only borderline significant.

**Figure 2:**
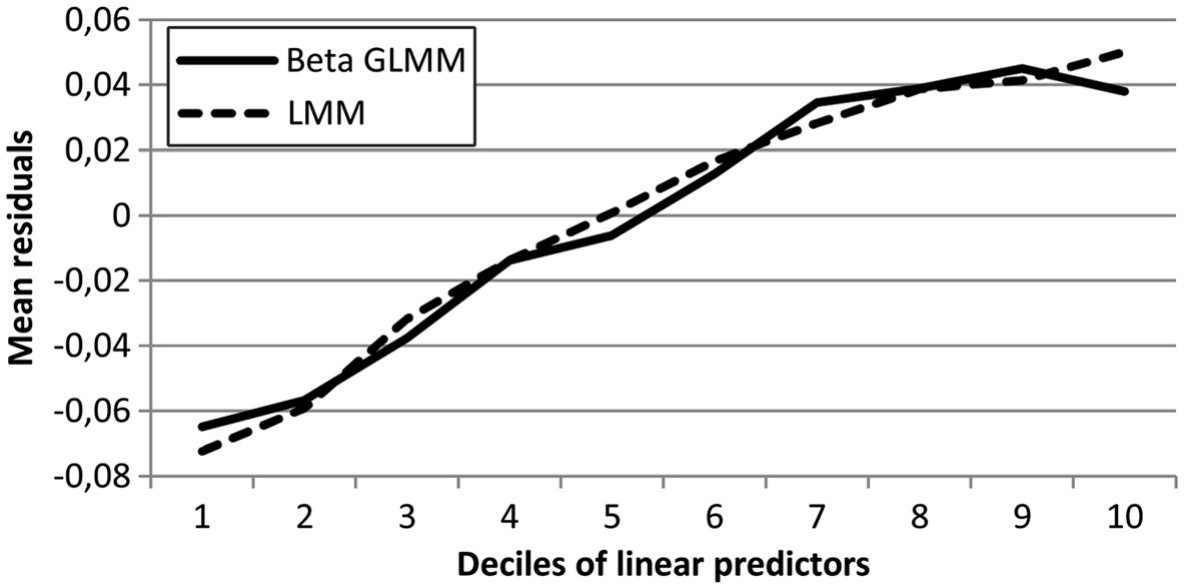
Mean residuals across deciles of linear predictors for beta GLMM and LMM in the KORA data.

Figure 2 shows the mean residuals across deciles of the linear predictors for the KORA data. One observes a strong correlation between residuals and predicted means for both LMM and beta GLMM, suggesting that both models overestimated the mean at the lower, and underestimated the mean at the upper part of the distribution. Probably, this results from the fact that generic HRQL scores are usually highly dispersed and that we only included very few covariates in our model.

Parameter coefficients from beta GLMM and beta GEE are difficult to compare, however, one recognizes that parameters in the GEE are estimated with less precision. The adjusted mean SF-6D scores from LMM and beta GEE model together with their 95% confidence intervals are shown in Table 2. It shows that both models produce very similar estimates but that for individuals with diabetes, standard errors from the LMM were slightly smaller than those from the beta GEE.

**Table 2:** Adjusted marginal mean SF-6D scores with 95% confidence intervals for time and diabetes in the KORA data (N = 1945)

**Time**		**T1**	**T2**
**Diabetes**	LMM	0.757 (0.732 – 0.782)	0.713 (0.692 – 0.735)
	Beta GEE	0.758 (0.729 – 0.784)	0.712 (0.689 – 0.735)
**No diabetes**	LMM	0.784 (0.776 – 0.792)	0.770 (0.760 – 0.780)
	Beta GEE	0.786 (0.778 – 0.794)	0.772 (0.761 – 0.781)

Effects of age and sex are set equal to their mean values.

*LMM* Linear mixed model, *GEE* Generalized estimating equations.

The regression models fitted to the ICF stroke data are shown in Table 3. Likewise, the table shows that the beta GLMM fitted the data better than the corresponding LMM. It achieved better AIC and BIC values and had a higher pseudo-R^2^. Furthermore, the beta GLMM respected the restricted range of the SIS-Mob scores, whereas 6 individual predictions based on the LMM estimates were lying outside the theoretically possible range. The significant interaction term between time and phase indicates that individuals in phase C showed greater improvement over time than individuals in phase D.

**Table 3:** Parameter estimates of LMM, beta GLMM and beta GEE in the ICF stroke data (N = 517)

**Parameter coefficients**	** LMM**		** Beta GLMM**		** Beta GEE**	
	**Estimate**	**p value**	**Estimate**	**p value**	**Estimate**	**p value**
Intercept	0.4955	<0.0001	−0.0106	0.9569	−0.0766	0.6828
Age (centered)	−0.0031	0.0060	−0.0226	0.0015	−0.0162	0.0137
Male sex	0.0528	0.0599	0.2931	0.0991	0.3200	0.0574
Time 2	0.0801	0.0003	0.4637	0.0005	0.3343	0.0010
Time 3	0.1597	<0.0001	0.9254	<0.0001	0.6792	<0.0001
Phase D	0.2941	<0.0001	1.6160	<0.0001	1.4316	<0.0001
Phase D*Time2	−0.0463	0.0807	−0.2005	0.2240	−0.0832	0.5154
Phase D*Time 3	−0.1178	<0.0001	−0.5465	0.0017	−0.3573	0.0477
σ^2^	0.0126					
ϕ			10.80			
**Variance of random effects**	Estimate	SE	Estimate	SE	Estimate	SE
Variance	0.0325	0.0038	1.2782	0.1632		
**Covariance estimates**						
Variance					0.0764	0.0061
Compound symmetry					0.1928	0.0230
Scale					0.0514	
**Fit statistics**						
−2LogL	−396.4		−924.1		-	
AIC	−376.4		−904.1		-	
BIC	−343.6		−871.3		-	
Pseudo-R^2^†	0.2277		0.7217		-	

*LMM* Linear mixed model, *GLMM* Generalized linear mixed model, *GEE* Generalized estimating equations, *SE* Standard error, *CS* Compound Symmetry, *AIC* Akaike information criterion, *BIC* Bayesian information criterion.

†Compared to linear random-intercept model with -2LogL = −262.8.

**Figure 3:**
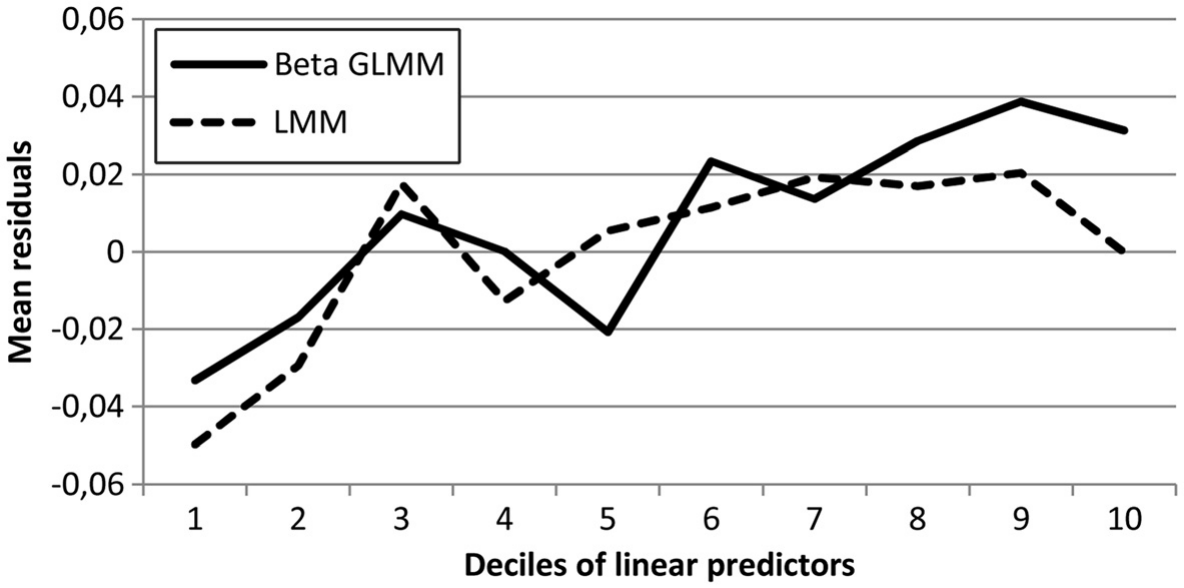
Mean residuals across deciles of linear predictors for beta GLMM and LMM in the ICF stroke data.

Looking at the mean residuals across deciles in Figure 3, one recognizes that, compared to the LMM, the beta GLMM underestimated the mean at the upper part of the distribution.

The adjusted mean SIS-Mob scores from LMM and beta GEE are shown in Table 4, suggesting that, again, both methods lead to nearly identical mean estimates. For patients in phase C, standard errors from the LMM were smaller than those from the beta GEE model, however, the opposite was true for patients in phase D.

**Table 4:** Adjusted marginal mean SIS-Mob scores with 95% confidence intervals for time and rehabilitation phase in the ICF stroke data (N = 517)

**Time**		**T1**	**T2**	**T3**
**Phase C**	LMM	0.521 (0.468 – 0.574)	0.601 (0.545 – 0.657)	0.681 (0.624 – 0.737)
	Beta GEE	0.520 (0.440 – 0.600)	0.602 (0.526 – 0.673)	0.681 (0.604 – 0.749)
**Phase D**	LMM	0.815 (0.779 – 0.852	0.849 (0.812 – 0.886)	0.857 (0.812 – 0.886)
	Beta GEE	0.819 (0.787 – 0.847)	0.853 (0.824 – 0.878)	0.862 (0.829 – 0.889)

Effects of age and sex are set equal to their mean values.

*LMM* Linear mixed model, *GEE* Generalized estimating equations.

The use of different values ε to move observations away from the boundary points in the beta GLMM did not appreciably affect parameter estimates; solely transformation (2) decreased the precision of estimates by about 20%.

## Discussion

Beta regression is a promising method for modeling HRQL data in cross sectional research [3,10], and recent methodological work has extended the beta regression model to deal with dependent observations [8,16]. In this paper, we examined the potential of beta regression methods in the analysis of longitudinal HRQL data. We highlighted the need to distinguish between mixed and marginal models, namely beta GLMM and beta GEE, when beta regression is extended to the longitudinal case. Using two empirical applications with data distributions typically encountered in practice, we compared the performance of the beta regression methods to that of the commonly used LMM.

Data collected in longitudinal designs typically have correlated observations, violating a basic assumption of ordinary regression methods. Longitudinal analyses require regression techniques that account for this dependence. In general, the correlation among repeated measures can be modeled implicitly, i.e. by including random effects as in the mixed model, or explicitly, i.e. by specifying a covariance structure between observations as in the marginal model. Through the inclusion of random effects, mixed models assume natural heterogeneity across individuals in some regression coefficients [18]. Random effects can also be motivated as an omitted subject-varying covariate, thus they give a potential explanation for the sources of correlation [19]. In contrast, marginal models treat the dependence between observations as nuisance and account for its effects by specifying a working correlation.

For linear longitudinal models, regression coefficients have the same interpretation regardless of how the correlation is modeled. For regression models with non-identity link such as beta regression, however, interpretation depends on whether a mixed model (i.e. a GLMM) or a marginal model is fitted. In the GLMM, estimated effects are adjusted for individual difference and thus only refer to within-individual change. In the marginal model, in contrast, the mean response is conditional only on covariates and not on other responses or random effects [18].

The choice between the two depends mainly on the specific scientific question of interest. GLMMs are most useful for making inferences about individuals and tracking individual trajectories, while the marginal model is more useful for inferences about population or sub-population averages. No model is a priori more suitable for the analysis of HRQL data than the other. It has been argued that mixed models may be more appropriate in epidemiological research as they allow a better understanding of the underlying mechanisms [28]. Also, they have a close relationship to matched-pair design methods often used in epidemiologic and public health research [19]. Due to the individual-specific interpretation of regression coefficients, the GLMM is also most meaningful for time-varying covariates. In contrast, the interpretation of time-invariant or between-subject covariates in the GLMM is less intuitive or even misleading since they also only allow a within-subject interpretation which is difficult to imagine. For example, if a beta GLMM is used to estimate treatment effects on HRQL in clinical trials, the respective treatment arm coefficient is interpreted as the difference in outcomes between two individuals with the same covariate values and the same random effects *b*_*i*_, differing only in their treatment arm. It does not describe the average treatment effect which is usually of major interest in intervention studies, especially if preference-based HRQL measures are used in economic evaluation studies [4]. Therefore, the marginal model may be more suitable in many applications in public health research. Also, it has been argued that many epidemiologic methods such as stratified methods are essentially population-averaged methods [19]. For our empirical applications this means that the change in SF-6D index scores associated with diabetes in the KORA data may be better described by a beta GLMM, while the difference in mean SIS scores between rehabilitation phases in the ICF stroke data may be better assessed using a beta GEE. Differences between beta GLMM and beta GEE also exist with respect to the handling of missing data: In practice, the beta GLMM may be more convenient since it remains valid under the MAR assumption which is usually more plausible in quality of life studies than the MCAR assumption made by the beta GEE.

A common approach to compare regression models and assess goodness of fit is to consider likelihood-based statistics which evaluate the probability of the observed data under the model. In both of our empirical examples, beta GLMM had better fit statistics (such as AIC, BIC or pseudo-R^2^) than the commonly used LMM, indicating that the beta distribution better accounted for the bounded support of the observed HRQL scores and their highly skewed distributions. However, an important question is whether better likelihood statistics make the beta GLMM more suitable than the LMM in practice. A similar issue has also been addressed previously: Zimprich used a beta GLMM to analyze longitudinal data on complex choice reaction time and concluded from better likelihood-based fit statistics that beta GLMM fitted the data much better than a LMM did [17]. However, given a fairly close similarity between parameter estimates, he also raised the question whether apart from these statistical considerations, beta GLMM is worth the effort to apply in practical data analyses. We even go one step further arguing that the likelihood may not be the most relevant criterion when comparing models to analyze HRQL data. Distributional fit and predicted densities may be important in applications with focus on individual density forecasts, such as in the reaction time example. However, when analyzing HRQL data in RCTs or cohort studies, conditional means rather than predictive densities are commonly of major interest [4]. Against this background, more attention should be attached to the question whether the mean structure is appropriately reproduced by the model. Figure 2 showed that the beta GLMM reproduced the observed values at the upper end of the distribution less satisfactorily than the LMM. This may be explained by the fact that beta regression fits both means and variances to the data. Since in the beta distribution the variance is a function of the mean, the estimated mean function may be biased. This phenomenon has already been observed in a cross-sectional design and suggests that full likelihood-based beta regression methods should be used with care when analyzing HRQL [3].

In the marginal perspective, beta GEE produced nearly identical estimates to the LMM, however, differences could be observed with respect to standard errors, especially in the ICF stroke data (Table 4). The larger standard errors of the beta GEE for patients in phase C are probably due to the fact that beta GEE provides robust standard errors using the sandwich formula. The smaller standard errors for patients in phase D, however, indicate that beta regression accounts for heteroscedasticity related to the bounded nature of the response variable [8]. This is because the predicted means of individuals in phase D were rather high, and for outcomes bounded on the unit interval, the variability of scores declines as the mean approaches one.

An important limitation of the beta regression is that it does not contain the boundary points 0 and 1 so that quite arbitrary transformation methods need to be applied. However, our sensitivity analyses support previous research in that parameter estimates are robust to the choice of transformation, provided that the values are moved far enough away from the boundaries.

The two empirical data sets used in this study were chosen to cover different types of studies commonly encountered in HRQL research. In particular, we addressed both disease-specific and generic HRQL scores and used data both from a cohort study and from a clinical setting. The two illustrative examples tackle clinically relevant research questions that have also been addressed in other studies [44-46]. However, since this paper focused on the comparison between different methodological approaches, we did not deal in detail with interpreting results in the healthcare context. For the purpose of this paper we have also made some simplifications, e.g. we did not consider model building but preferred using a rather lean model with only a few covariates. Also, we treated the precision parameter in the beta GLMM as constant instead of modeling it in terms of covariates, although such an approach may have improved model fit [10,17,26].

Another limitation of our study is that our empirical data only provided up to three measurements per individual. Further research is needed to examine the use of beta regression in more complex study designs. Also, we did not consider random slopes which are commonly used to model heterogeneity in the effect of time on the response variable [15]. However, for reasons of model convergence, it is not recommended to fit anything more complex than a single random intercept model to non-normal data with only a few time points per person. Similarly, we did not consider working correlation structures other than compound symmetry in the beta GEE. However, compound symmetry assumes the same correlation for all observations within a person which we think is reasonable in the case of only a few time points per person. Furthermore, it corresponds to the correlation structure implicitly modeled by the mixed model with single random intercept.

## Conclusions

In conclusion, longitudinal beta regression models are a natural candidate to analyze HRQL over time since they account for the bounded range and the skewed distribution of the response variable. However, depending on whether a population-averaged or a subject-specific approach is preferred, researchers should distinguish between a mixed (beta GLMM) and a marginal (beta GEE) model. The mixed model may be more appropriate in cohort studies in order to track individual HRQL trajectories, while the marginal model is more suitable to estimate average treatment effects in intervention studies. Although beta regression addresses the specific idiosyncrasies of bounded HRQL data, empirical estimates only slightly differed from those of the commonly applied linear mixed model.

## Competing interests

The authors declare that they have no competing interests.

## Authors’ contributions

MH devised the concept for the paper, performed the statistical analysis, interpreted the data and drafted the manuscript. AD was involved in the coordination of the KORA study and commented on drafts of paper. RH was involved in the conception of the KORA study and assisted in writing the manuscript. All authors read and approved the final manuscript.

## Pre-publication history

The pre-publication history for this paper can be accessed here:

http://www.biomedcentral.com/1471-2288/12/144/prepub

## Supplementary Material

Additional file 1 SAS Code used to fit LMM, beta GLMM and beta GEE to the KORA data. Click here for file
