# Supplementary material for: Longitudinal beta regression models for analyzing health-related quality of life scores over time
Source: BMC Med Res Methodol. 2012 Sep 17;12:144. doi: 10.1186/1471-2288-12-144 (PMC3528618; doi:10.1186/1471-2288-12-144)
Supplement: Additional file 1 — SAS Code used to fit LMM, beta GLMM and beta GEE to the KORA data. [file 1471-2288-12-144-S1.pdf]

SAS-Code for linear mixed model, mixed beta model (beta GLMM) and beta GEE fitted to the KORA data

Dependent variable is the SF-6D index (sf6d). Covariates are centered age at baseline (age_cent), time, sex, and diabetes (diab). The model also includes the interaction between diabetes and time.

```
*Linear mixed model;
proc glimmix data=data_kora method=quad;
class id_nr time sex diab;
model sf6d = age_cent time sex diab time*diab / dist=gaussian s ddfm=bw;
random intercept / subject=id_nr;
run;


*Mixed beta model;
proc glimmix data=data_kora method=quad;
class id_nr time sex diab;
model sf6d = age_cent time sex diab time*diab / dist=beta s ddfm=bw;
random intercept / subject=id_nr;
run;


*Beta GEE;
proc glimmix data=data_kora empirical;
class id_nr time sex diab;
model sf6d = age_cent time sex diab time*diab / dist=beta s ddfm=none covb;
random _residual_ / subject=id_nr type=cs vcorr;
lsmeans diab*time /ilink cl;
run;
```
